# Functional modulation of human monocytes derived DCs by anaphylatoxins C3a and C5a

**DOI:** 10.1016/j.imbio.2011.07.033

**Published:** 2012-01

**Authors:** Ke Li, Henrieta Fazekasova, Naiyin Wang, Qi Peng, Steven H. Sacks, Giovanna Lombardi, Wuding Zhou

**Affiliations:** a King's College London, MRC Centre for Transplantation, NIHR Comprehensive Biomedical Research Centre, Guy's Hospital, London, UK; b Xi’an Jiaotong University, Core Research Laboratory, The Second Affiliated Hospital, School of Medicine, Xi’an, China

**Keywords:** Anaphylatoxins, C3a, C5a, Dendritic cells, Functional modulation, T cell response

## Abstract

Anaphylatoxins C3a and C5a are important modulators for dendritic cell activation and function in mice. In order to verify the significance of these observations in man, we have investigated the functional modulation of human monocytes derived DCs by C3a and C5a. Here we report that engagement of C3aR or C5aR on human monocytes derived DCs (moDCs) enhances the cell activation and their capacity for allostimulation. In addition, we show that intracellular production of cAMP is reduced and PI3K/AKT, ERK and NF-κB signalling is increased following stimulation with C3a or C5a, identifying intracellular signalling pathways that could convert cell surface C3aR and C5aR engagement into changes in moDC functions. Our data provide evidence that human DCs are equipped to react to C3a/C5a and undergo phenotypic change as well as functional modulation. Complement offers a potential route to modulate human DC function and regulate T cell mediated immunity.

## Introduction

The small fragments (e.g. C3a and C5a) released during the course of complement activation play an important role in inflammation. They act on specific receptors (e.g. C3aR and C5aR) expressed on different types of cells (e.g. mast cells, endothelial cells, and phagocytes) causing cell activation and degranulation, and histamine release, which in turn increase vascular permeability, extravasation of serum proteins and migration of phagocytes, thus contributing to local inflammation. Besides their traditional roles in inflammatory responses, it has now become clear that anaphylatoxins (C3a, C5a) also play important roles in regulating the adaptive immune responses (reviewed by Peng et al. 2009a; Klos et al. 2009). One important mechanism underlying this regulation is that C3a/C5a acts on their receptors expressed on antigen presenting cells such as dendritic cells, monocytes/macrophages to modulate the cell activation and function, consequently regulating antigen specific T cell responses.

Recent studies in mouse have demonstrated that C3a and C5a can regulate T cell responses through modulating DC activation and function. It has been shown that stimulation of C3aR with receptor ligand (C3a) increased the capacity of murine bone morrow (BM) DC for Ag presentation and enhanced its ability to stimulate the allospecific T cell response. Conversely, in the absence of C3a–C3aR interactions, due to absent expression of C3aR, DC function was impaired, and manifested as a less activated phenotype, with reduced capacity for Ag uptake/presentation and lowered ability to stimulate Th1 responses *in vitro* and *in vivo* (Li et al. 2008; Peng et al. 2008). Similar observations have being made on C5a, C5a stimulation enhanced murine BMDC activation and its function in allostimulation (Peng et al. 2009b). Conversely BMDCs from C5aR deficient mice had less-activated phenotype with lowered surface expression of MHC class II, CD86 and CD40, and consequently had reduced capacity to stimulate allospecific T cells. In support for the role for C5a in DC functional modulation, BMDCs from C5 deficient mice (which are unable to generate C5a) produced less IL-12 (a Th1 driving molecule) and expressed lower CD40 after BCG infection compared with DCs from WT mice; BCG-primed C5 deficient DCs elicited defective Th1 responses, concurrent with an increased growth of BCG in lungs (Moulton et al. 2007). These results provide strong evidence that C3a and C5a can act directly on their receptors expressed on murine DCs resulting in cell activation and functional modulation, consequently up-regulating Th1 responses.

So far, most evidence for C3a/C5a acting on DCs comes from the studies in mice. Such phenomenon has not been well documented in human DCs. A previous study on a single C3 deficient person has found that the *in vitro* development of immature dendritic cells and their maturation capacity were greatly impaired, with decreased CD1a expression and IL-12p70 secretion ability, suggesting that complement activation plays an important role in DC differentiation and maturation, which is possibly through the effect of complement effectors (e.g. C3a and C5a) (Ghannam et al. 2008). Furthermore, several studies on human monocyte-derived DCs (moDCs) have shown that DCs express a wide range of complement components, receptors and regulators, including C3aR and C5aR (Weinmann et al. 2003; Li et al. 2011; Gutzmer et al. 2004); C3a or C5a stimulation induces F-actin polymerisation and up-regulates the surface expression of CD54, CD83 and CD86 in DCs (Weinmann et al. 2003; Gutzmer et al. 2004), suggesting that DCs can detect and respond to C3a anf C5a stimulation. However, the functional significance of these observations, in terms of modulating DC function and regulating T cell responses is not well defined.

In the present study, we investigated the expression of surface molecules, cytokine profile and function of human moDCs in response to C3a or C5a stimulation. We also explored the signalling pathways that are involved in C3a/C5a mediated DC modulation.

## Materials and methods

### Reagents

Microbeads for CD14 were purchased from Miltenyi (Surry, UK). GM-CSF was purchased from Firstlink (Brierley Hill, UK). Recombinant human C5a was purchased from Sigma–Aldrich (Dorset, UK). Purified human C3a was purchased from Cortex Biochem (CA, USA). [^3^H]-TdR and ECL Western Blotting Detection Reagents were purchased from GE Healthcare UK Limited (Bucks, UK). Antibody reagents used in signalling pathway studies were purchased from Cell Signalling Technology (Beverly, USA). Cytometric beads array (CBA) kit was purchased from BD Biosciences (Oxford, UK). The following monoclonal anti-human antibodies were used for the detection of cell surface molecule and complement receptor expression by flow cytometry: FITC anti-HLA-DR (Sigma, St. Louis, USA), -CD40 (BD Biosciences, Oxford, UK), -CD86 (Caltag Medsystem, Buckingham, UK); PE anti-C3aR, -C5aR (Biolegend, San Diego, USA).

### Samples

Human buffy coats were obtained from NBS-South Thames, London, UK, which was approved by the local research ethics committee of Guy's Hospital, London, UK.

### Preparation of human DCs

Monocyte-derived DCs were generated from peripheral blood monocytes by treatment with GM-CSF and IL-4. PBMC were isolated from buffy coat preparations from healthy donors by Ficoll-Hypaque gradient centrifugation, followed by anti-CD14 bead selection. The CD14 negative cell fraction was used for T cell isolation, as described below. CD14^+^ monocytes were cultured in RPMI-10% FCS, 2 mM l-glutamine, and 100 U/ml penicillin and streptomycin in the presence of GM-CSF (20 ng/ml) and IL-4 (20 ng/ml) at 37 °C in 5% CO_2_ atmosphere for 5 days. Cells were further cultured for 24 h with or without C3a or C5a at different concentrations, unless otherwise specified.

### Preparation of T cells

T cells were prepared from the CD14^−^ fraction of human buffy coats. CD4^+^ T cells were obtained by negative selection using anti-CD8, anti-CD33, anti-CD16, anti-CD19 and anti-CD56.

### RT-quantitative PCR

Real-time RT-quantitative PCR (RT-qPCR) was performed with an MJ Research PTC-200 Peltier Thermal Cycler and DyNAmo™ HS SYBR^®^ Green qPCR kit (MJ BioWorks, Finland). PCR was set up in low-profile microplates containing 10 μl of master mix, 1 μl diluted cDNA (reflecting 0.1 μg of total RNA), 5 pmol of each 3′ and 5′ primer pair, either for each testing gene or 18s gene (the information for primer sequences are shown in Supplementary table), in 20 μl reaction volume. Amplification was performed according to manufacturer's cycling protocol and done in triplicate. Gene expression was expressed as 2^−ΔΔ(Ct)^, where Ct is cycle threshold, ΔΔ(Ct) = un-stimulated DCs Δ(Ct) − C3a or C5a stimulated DCs Δ(Ct); Δ(Ct) = testing gene (Ct) − 18s (Ct).

### Measurement of intracellular cAMP

DCs (2 × 10^6^) were seeded in 12 well plate and cells were incubated with forskolin (5 nM) for 30 min. The cells were further incubated for 30 min in the presence or absence of C3a or C5a. The treated cells were re-suspended cells in cell lysis buffer (R&D Systems, Europe Ltd, Abingdon, UK). Cell lysate was centrifuged for 10 min at 600 × *g* to remove cellular debris. The concentration of cAMP was determined in each sample using a Parameter™ cAMP assay kit (R&D Systems) according to the manufacturer's instructions.

### Measurement of cytokine production

IL-12 production was measured in supernatants from C3a or C5a treated DCs in the presence of CD40L-transfected L cells by sandwich ELISA. The purified and biotinylated antibody pairs were purchased from BD Biosciences, UK. The production of IL-6, and TNF-α was measured in the supernatants of DC cultures, and IFN-γ, TNF-α and IL-17A were measured in the supernatants of DC/T cell co-cultures by CBA assays.

### Analysis of DC allostimulatory capacity *in vitro*

To assess the stimulatory capacity of DCs, irradiated DCs (1 × 10^4^ per well) were co-cultured with purified allogeneic CD4^+^ T cells (3 × 10^4^/well). After 5 days, cell cultures were pulsed with 1 μCi/well [^3^H]-TdR, and the proliferation was measured as [^3^H]-TdR incorporation after 20 h incubation.

### Western blot analysis

6 day DCs (2 × 10^6^) were stimulated with C3a or C5a for up to 30 min. Cells were washed and lysed in 200 μl of lysis buffer (Perbio Science, Northumberland, UK). Prior to analysis, lysates were homogenized with an ultrasonic cell disruptor and cleared by centrifugation (20 min, 15,000 × *g* at 4 °C). 10 μl extract (corresponding to 1 × 10^5^ cells) per lane was separated onto 12% SDS PAGE followed by electro blotting. Blocking was performed in Tris-buffered saline (TBS) containing 5% non-fat milk power for 1 h. Membranes were incubated with the following primary antibodies in TBS containing 5% BSA and 0.1% Tween 20 overnight at 4 °C: anti-phospho-ERK1/2, -AKT and -IκB antibodies and anti-ERK1/2, -AKT and -IκB antibodies. Immunoblots were developed using ECL Western Blotting Detection Reagents. The relative amounts of phosphorylated proteins were evaluated through densitometry using Image J software (NIH Image).

## Results

### Phenotypic changes of DCs in response to C3a or C5a stimulation

**Fig. 1:**
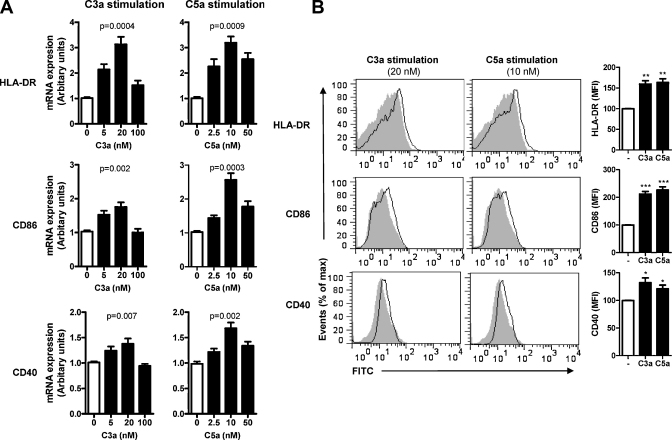
Effects of C3a and C5a stimulation on the expression of HLA-DR, CD86 and CD40 by moDCs. DCs were treated with C3a or C5a at different concentrations for 24 h and then used for analyzing the gene expression by RT-PCR and cell surface expression by flow cytometry. (A) Gene expression of HLA-DR, CD86 and CD40. Data are presented as means ± SEM of 3 individual experiments. Statistical significance calculated using one-way ANOVA. (B) Surface expression of HLA-DR, CD86 and CD40. The left panel shows representative histogram plot overlay of the un-stimulated DCs (gray and black profiles) and C3a or C5a stimulated DCs (open profiles). The right panel displays mean fluorescence intensity (MFI) of each surface molecule. Data are presented as percentage of MFI of un-stimulated DCs, set as 100%. Data are means ± SEM of 3 independent experiments. Statistical significance calculated using Student's *t*-test (*p < 0.05, **p < 0.01, ***p < 0.005) compared with un-stimulated DCs.

One of the most important functions for DC is the stimulation of T cells, and this largely depends on their state of activation, we initially investigated the phenotypic changes of human moDCs in responses to C3a or C5a stimulation. We treated moDCs with either C3a or C5a at different concentrations for 24 h, and analyzed the gene and cell surface expression of MHC class II and co-stimulatory molecules by RT-PCR and flow cytometry. We found that gene expression of HLA-DR, CD86 and CD40 in DCs was increased by C3a or C5a stimulation, particularly at relatively low concentrations (C3a: 5–20 nM, C5a: 2.5–10 nM). The gene expression was less affected at the high concentration of C3a (100 nM) or C5a (50 nM) (Fig. 1A). Consistent with the gene expression, cell surface expression of HLA-DR, CD86 and CD40 was increased by C3a or C5a stimulation (Fig. 1B). These results indicate that DCs exhibit a more activated phenotype following C3a or C5a stimulation at certain concentrations.

### Effects of C3a or C5a stimulation on inflammatory cytokine production by DCs

**Fig. 2:**
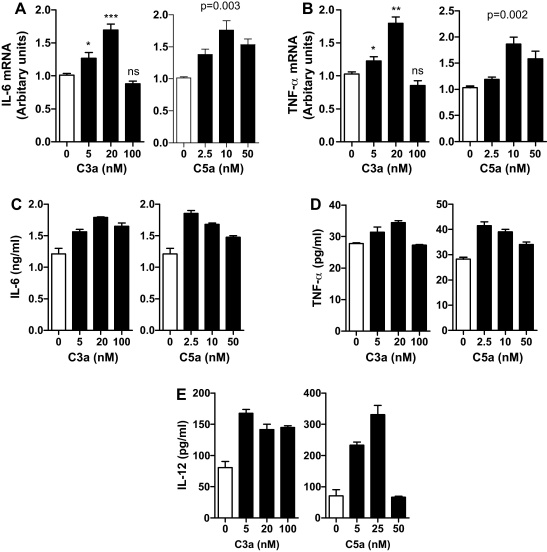
Effects of C3a and C5a stimulation on cytokine production by moDCs. DCs were treated with C3a or C5a at different concentrations for 24 h. The cells and the supernatants were used for analyzing the gene expression and cytokine secretion. (A and B) Gene expression of IL-6 and TNF-α. Data are presented as means ± SEM of 3 individual experiments. Student's *t*-test or ANOVA was used where appropriate to determine significant differences between un-stimulated and C3a or C5a stimulated DCs (*p < 0.05, **p < 0.01, ***p < 0.005). (C and D) Secretion of IL-6 and TNF-α. (E) Secretion of IL-12. Data in C–E are presented as means ± SEM of duplicate samples and are representative of 3 individual experiments.

Having shown that both C3a and C5a have effects on DC activation phenotype, we next examined the effects of C3a or C5a stimulation on inflammatory cytokine production by DCs. We treated moDCs with either C3a or C5a at different concentrations for 24 h, and analyzed the gene expression and cytokine secretion of IL-6 and TNF-α by the cells. We found that gene expression of IL-6 and TNF-α was increased by C3a or C5a stimulation in a dose dependent manner (at 0–20 nM for C3a and 0–10 nM for C5a), though the high dose of C3a (100 nM) had no effect on both IL-6 and TNF-α gene expression (Fig. 2A and B). In addition to the changes of gene expression, protein levels of IL-6 and TNF-α in 24-h supernatants of DCs were also increased in the presence of C3a or C5a, though the effect of C3a on TNF-α was relatively small (Fig. 2C and D). We also examined the effect of C3a and C5a on CD40L mediated IL-12 production by DCs and found that C3a or C5a pretreated DCs produced significantly higher amount of IL-12 compared with untreated DCs (Fig. 2E). Together, these results indicate that DCs have increased ability to produce inflammatory cytokines in response to C3a/C5a stimulation. It also suggests that following the C3a/C5a stimulation, DCs become more potent to stimulate Th1 responses, as a result of up-regulated IL-12 production.

### Effects of C3a and C5a stimulation on the expression of C3aR and C5aR by moDCs

**Fig. 3:**
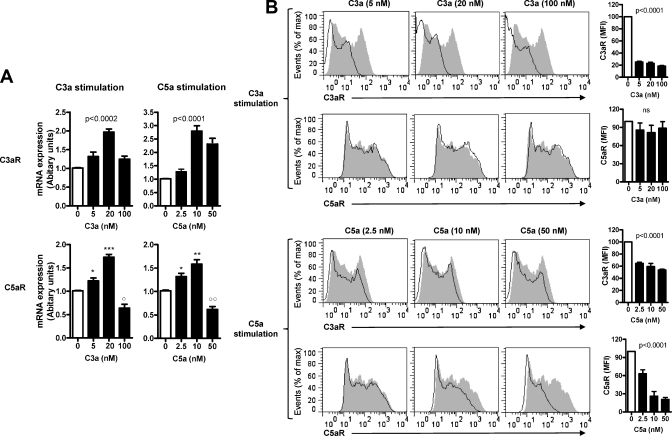
Effects of C3a and C5a stimulation on the expression of C3aR and C5aR by moDCs. DCs were treated with C3a or C5a at different concentrations for 24 h and then used for analyzing the gene by RT-PCR and cell surface expression of C3aR and C5aR by flow cytometry. (A) Gene expression of C3aR and C5aR. (B) Surface expression of C3aR and C5aR. Histogram plot overlay of the un-stimulated DCs (gray and black profiles) and C3a or C5a stimulated DCs (open profiles) are shown in the left panel and the quantification of MFI is shown in the right panel. Data in A and B are presented as means ± SEM of 3 individual experiments. Student's *t*-test or ANOVA was used where appropriate to determine significant differences between un-stimulated and C3a or C5a stimulated DCs (*p < 0.05; **p < 0.01, ***p < 0.005, ns, not significant).

Our previous study with moDCs has shown that gene expression of C3aR and C5aR can be regulated by human serum which contains complement, suggesting that complement activation itself can lead to auto-regulation of C3aR and C5aR expression. To investigate the possibility that C3a/C5a could auto-regulate the expression of C3aR and C5aR by DCs, we treated moDCs with either C3a or C5a at different concentrations for 24 h, and analyzed the gene and cell surface expression of C3aR and C5aR by RT-PCR and flow cytometry. We found that the gene expression of both receptors was up-regulated by lower doses of C3a (5 and 20 nM) and C5a (2.5 and 10 nM), this was consistent with the observations on other molecules (i.e. MHC class II and co-stimulatory molecules, IL-6, TNF-α) (Fig. 3A). Interestingly, however, we found that cell surface expression of both receptors was reduced following the stimulation with its specific ligand in a dose dependent manner (C3a: 0–100 nM, C5a: 0–50 nM) (Fig. 3B). Thus, C3a or C5a stimulation up-regulated the gene expression of both C3aR and C5aR, but down regulated the cell surface expression of its own receptor in moDCs. In addition, C5a stimulation also caused a small reduction in cell surface expression of C3aR, but C3a stimulation had no effect on surface expression of C5aR (Fig. 3B).

### Effects of C3a and C5a on allo-stimulatory ability of moDCs

**Fig. 4:**
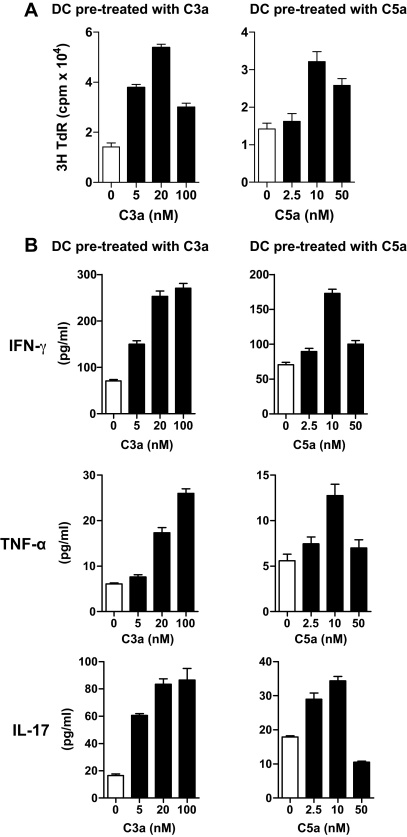
Effects of C3a and C5a on allo-stimulatory ability of moDCs. DCs were treated with C3a or C5a at different concentrations for 24 h and then co-cultured with purified allogeneic CD4 T cells. Proliferative responses and cytokine secretion were assessed by thymidine uptake (at 72 h) and CBA assay (120 h), respectively. (A) T cell proliferation. Data are presented as means ± SEM of triplicate samples and are representative of 4 individual experiments. (B) Cytokine secretion. Data are presented as means ± SEM of duplicate samples and are representative of 4 individual experiments.

So far we have demonstrated the effects of C3a and C5a on moDC activation phenotype and inflammatory cytokine production; we next investigated whether C3a and C5a can modulate moDC function in T cell stimulation. We pre-treated moDCs with either C3a or C5a at different concentrations for 24 h, and then co-cultured them with purified allogeneic CD4 T cells. The cell proliferative response and inflammatory cytokine production were carried out after 3 and 5 days of co-culture, respectively. We found that there were higher proliferative responses when T cells were co-cultured with DCs pre-treated with C3a or C5a compared with those co-cultured with untreated DCs (Fig. 4A). Furthermore, the levels of several inflammatory cytokines including IFN-γ, IL-17A and TNF-α were increased in the supernatants of co-cultures of T cells and C3a or C5a pre-treated DCs, compared with co-cultures of T cells and untreated DCs (Fig. 4B). The enhancement of proliferative responses and inflammatory cytokine production appear to occur in a dose-dependent manner for C3a (0–20 nM) and C5a (0–10 nM). A relatively high dose of C5a (50 nM) has less effect or even adverse effect in some cases. Together, these results suggest that engagement C3aR or C5aR on DCs has a positive impact on DC activation and its function in T cell stimulation.

### C3a or C5a stimulation mediates inhibition of cAMP production in moDCs

**Fig. 5:**
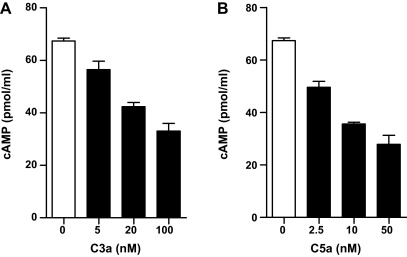
C3a or C5a stimulation mediates inhibition of cAMP production in moDCs. (A and B) DCs were pretreated with forskolin for 30 min and then treated with C3a or C5a at different concentrations for 30 min. cAMP levels were measured in the cell lysates. Data are presented as means ± SEM of duplicate samples and are representative of 3 individual experiments.

Having demonstrated roles for C3aR and C5aR in regulating DC activation and function in T cell stimulation, we next examined the intracellular signalling pathways by which C3aR and C5aR could elicit such effects. C3aR and C5aR are G-protein coupled receptors (GPCRs). GPCRs can activate multiple intracellular signalling pathways, among which cAMP pathway is one of the key pathways. cAMP is a cyclic nucleotide that functions as an intracellular second messenger which regulates a wide range of important cellular processes. It has been shown that elevated cAMP has a negative impact on DC activation and function (Galgani et al. 2004; Kambayashi et al. 2001). We have previously shown that ligation of C3aR or C5aR on murine BMDCs induced inhibition of cAMP production, consequently permitting DC activation (Li et al. 2008; Peng et al. 2009b). Therefore, we investigated whether the same is true for human moDCs. We stimulated moDCs with C3a or C5a at different concentrations and measured the cAMP in the cell lysate. We found that C3a or C5a stimulation reduced the cAMP levels in moDCs in a dose dependent manner (Fig. 5). Thus, our results, in consistence with murine BMDCs, demonstrate that engagement of C3aR or C5aR on human moDCs induces inhibition of cAMP production, which consequently may release the DC from the inhibitory effects of cAMP and thereby up-regulate DC activation and its functions.

### C3a or C5a stimulation activates PI3K, MAPKs and NF-κB signalling in moDCs

**Fig. 6:**
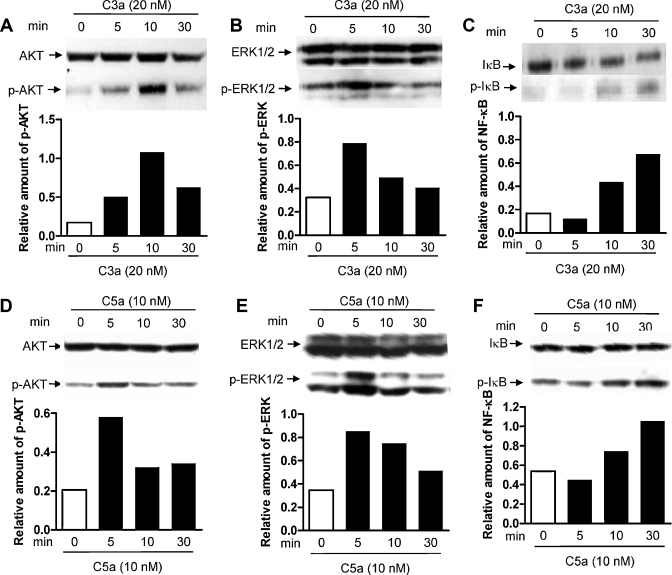
C3a or C5a stimulation activates PI3K, MAPKs and NF-κB signalling in moDCs. DCs were stimulated with an optimized dose of C3a (20 nM) (A–C) or C5a (10 nM) (D–F) for indicated time periods. The cell lysates ware used for analyzing phosphorylation of AKT, ERK1/2, and IκB by Western blot. In each blot the top row of bands corresponds to incubating membrane with appropriate total antibody and the bottom row of bands corresponds to incubating membrane with appropriate anti phospho-antibody. Relative amounts of p-AKT, p-ERK1/2 and p-IκB are shown in the lower panel of the each figure. A representative of 3 independent experiments is shown.

Besides cAMP, we investigated several other important signalling pathways (PI3K, MAPKs and NF-κB) which are also known to be activated downstream of GPCR ligation (Marinissen and Gutkind 2001), to determine their role in converting C3aR and C5aR signalling into DC functional change. We examined phosphorylation of IκBα (an indicator of NF-κB activation), AKT (a downstream effector of PI3K) and ERK1/2 (a member of MAPKs) in response to C3a or C5a stimulation. We found that phosphorylation of AKT (p-AKT), ERK (p-ERK) and IκBα (p-IκBα) was detected in both un-stimulated and C3a or C5a stimulated moDCs. Following C3a (20 nM) stimulation, the amount of p-AKT, p-ERK and p-IκBα was increased and peaked at 10, 5 and 30 min, respectively (Fig. 6A–C). Following C5a (10 nM) stimulation, p-AKT, p-ERK and p-IκBα were similarly increased, except that p-AKT peaked (at 5 min) more rapidly than with C3a stimulation (Fig. 6D–F). These results indicate that engagement of C3aR or C5aR on moDCs can induce rapid activation of PI3K, ERK and NF-κB pathways, which could confer C3a or C5a mediated up-regulation of DC activation and functions.

## Discussion

The present study extends our previous findings in murine BMDCs to human moDCs by showing that the cell activation, inflammatory cytokine production and allo-stimulatory ability of moDCs were up-regulated by C3a or C5a at certain concentrations. Since C3a or C5a has similar effects in mouse and human DCs, this suggests a well conserved biological mechanism for strengthening and directing the adaptive immune response.

Using C3a or C5a to stimulate (relative immature) day 5 moDCs, we demonstrate that, C3aR or C5aR engagement up-regulates DC activation which is evident by increased expression of MHC class II and co-stimulatory molecules and inflammatory cytokine production (i.e. IL-12, IL-6, TNF-α). Moreover, C3aR or C5aR engagement up-regulates DC function in T cell stimulation which was supported by enhanced proliferative responses and Th1 cytokine (IFN-γ, TNF-α) production upon allostimulation. As direct allostimulation is largely dependent on MHC and co-stimulatory molecule expression on donor APCs, and in view of the fact IL-12 released by DCs can act as a T cell stimulating factor, C3a or C5a mediated modulation of these molecules could confer an enhanced allo-stimulatory capacity of DCs. Furthermore, our data show that in addition to Th1 cytokine production, Th17A was also elevated in the supernatants when T cells were co-cultured with C3a or C5a treated DCs compared with untreated DCs, such elevation could be attributed to enhanced IL-6 production by DCs following C3a or C5a stimulation (Acosta-Rodriguez et al. 2007). These data suggest that C3a and C5a have positive effect on both Th1 and Th17 responses in our system. However, given that the amount of IL-17A detected in our assays was relatively small compared with that of IFN-γ, and the secretion of IL-12 (a driver of Th1 responses) by DCs was significantly increased following C3a or C5a stimulation, it would suggest that modulation of allo-stimulatory ability of DCs by C3a or C5a mainly involves the regulation of Th1 responses.

We have used different ranges of C3a (0–100 nM) and C5a (0–50 nM) to assess their effects on DC activation and function. The choice of the higher dose range of C3a than that of C5a in our study was based on the fact that serum concentrations of C3a are higher than those of C5a under both normal and pathological conditions (Bengtson and Heideman 1988). Our data show that 0–20 nM of C3a or 0–10 nM of C5a was a concentration range within which moDC's activation, inflammatory cytokine production and allo-stimulatory ability were up-regulated. In some cases of our study, the 100 nM C3a or 50 nM C5a had less effect or even inhibitory effect on DC activation and function. Therefore, the regulation of DC function by C3a/C5a seems to depend on C3a/C5a concentrations which cells were exposed to; lower concentrations could have positive impact on cell activation and function, while higher concentrations could have negative impact on cells. In addition to the concentrations, C3a/C5a mediated DC regulation may also depend on the activation state of the cells. We have noted that more mature DCs, when exposed to C3a or C5a (even at lower doses), can either do not respond to the stimulation or have reduced cell activation and allo-stimulatory ability (our unpublished observation). Together, these observations highlight the complexities of the role for C3a and C5a in DC regulation, which would likely depend on the concentrations of C3a/C5a and the activation state of the DCs.

In this study we have investigated the effects of C3a or C5a on the expression of its own receptor by moDCs. Interestingly, we found that the cell surface expression was not correlated with the gene expression in response to C3a or C5a stimulation. The reasons for this were not clear and need to be further studied. We can postulate that the increased C3aR and C5aR gene expression could reflect the cell activation caused by either C3aR or C5aR engagement, while the reduced surface expression of the receptors could be explained by a phenomenon of GPCR called ligand induced receptor internalisation (Pierce et al. 2002). It is possible that DCs have a self control mechanism to prevent themselves from over activation by C3a or C5a, particularly under inflammatory/pathological conditions, through reducing cell surface expression of its own receptor (C3aR, C5aR). C5a stimulation also caused a small reduction in cell surface expression of C3aR, suggesting that C5a may involve C3aR internalisation.

Another important finding in this study is the identification of several intracellular signalling pathways by which C3a or C5a could modulate moDC function. First is intracellular cAMP, a potent negative regulator of inflammatory cytokines. Our previous studies in mouse have shown that elevation of intracellular cAMP in DCs is associated with lowered surface expression of MHC class II and co-stimulatory molecules, and decreased pro-inflammatory cytokine (i.e. IFN-γ, TNF-α, IL-12) production (Li et al. 2008; Peng et al. 2009b). In this study we extended our observations to human moDCs. Our results show that C3a or C5a stimulation significantly reduced intracellular levels of cAMP, and increased surface expression of MHC class II and B7.2 and IL-12 expression, demonstrating that engagement of C3aR/C5aR on DCs leading to inhibition of the adenylate cyclase pathway is important for modulating DC activation and function. In addition to the stimulation of conventional second-messenger-generating systems, such as cAMP, GPCRs also involve a complicated network of intracellular signalling pathways (e.g. PI3K, MAPKs and NF-κB). PI3K has been linked to an extraordinarily diverse group of cellular functions (e.g. cell growth, proliferation, differentiation, motility, survival and intracellular trafficking). MAPK/ERK is associated with cell division in many types of cells. NF-κB is a key transcription factor responsible for regulation of numerous genes during inflammation and immune responses. Our previous studies in DCs have shown that activation of PI3K, ERK1/2 and NF-κB mediates positive regulation of several cellular processes including antigen uptake, antigen presentation and pro-inflammatory cytokine production. In this study we show that engagement of C3aR/C5aR mediates the activation of PI3K, ERK and NF-κB pathways in human DCs, suggesting that C3a/C5a can exert its functions through C3aR/C5aR mediated signalling pathways. Thus our findings provide evidence that C3a/C5a can directly act on human DCs through interaction with their receptors on DCs, which induce a network of intracellular signalling pathways and subsequently modulate DC function.

In conclusion, in this study we demonstrate that human immature moDCs, in response to C3a or C5a stimulation, undergo phenotypic activation and functional modulation. Although some of the DC responses to C3a or C5a that we presented in this study are not extraordinarily large, all the responses were consistently observed in least 3 independent experiments and occurred in a dose dependent manner. We also show that engagement of C3aR or C5aR results in inhibition of cAMP pathway and activation of PI3K, MAPKs and NF-κB signalling pathways which could confer the up-regulated DC activation and function. The behavioural similarities of human and mouse DCs in response to complement activation products C3a/C5a provide a necessary translational step and strengthen the rationale for exploring complement based strategies for the manipulation of human immune mediated disease.
