# Supplementary material for: Functional modulation of human monocytes derived DCs by anaphylatoxins C3a and C5a
Source: Immunobiology. 2012 Jan;217(1):65–73. doi: 10.1016/j.imbio.2011.07.033 (PMC3234345; doi:10.1016/j.imbio.2011.07.033)
Supplement: Supplementary file 1 [file mmc1.doc]

**Supplemental Table**

# PCR primer sequences and product sizes

| Primer* | **Oligonucleotide Sequence**  **(5’ → 3’)** | **Product Size (bp)** | **Gene bank code** |
| --- | --- | --- | --- |
| HLA-DR-1 | agacaagttcaccccaccag | 379 | NM_019111.3 |
| HLA-DR-2 | tcacctccatgtgccttaca |  |  |
| CD86-1  CD86-2 | agacgcggcttttatcttca ggttgcccaggaacttacaa | 387 | NM_006889 |
| CD40-1  CD40-2 | CCTCGCTATGGTTCGTCTGCC AGCCAGGAAGATCGTCGGGA | 740 | AF199028 |
| IL-6-1 | aaagaggcactggcagaaaa | 351 | NM_000600 |
| IL-6-2 | aaagctgcgcagaatgagat |  |  |
| TNF-α-1 | tgcttgttcctcagcctctt | 514 | NM_000594 |
| TNF-α-2 | ggaagacccctcccagatag |  |  |
| C3aR-1  C3aR-2 | ACTCGTGGAGACATCCAGGT  GAAGATTTCCCGGTACACGA | 555 | NM_004054 |
| C5aR-1  C5aR-2 | GAGCCCAGGAGACCAGAACATG  TACATGTTGAGCAGGATGAGGGA | 441 | NM_001736 |
| 18S-1 | GACTCAACACGGGAAACCTC | 153 | NM_011296.1 |
| 18S-2 | ATGCCAGAGTCTCGTTCGTT |  |  |

* Primer-1 is identical to the coding strand; primer-2 is complementary to the coding strand. All primers were designed such that there are intronic sequences between the primer 1 and primer 2.
